# LINC00511 promotes the malignant phenotype of clear cell renal cell carcinoma by sponging microRNA-625 and thereby increasing cyclin D1 expression

**DOI:** 10.18632/aging.102156

**Published:** 2019-08-21

**Authors:** Huanghao Deng, Changkun Huang, Yinhuai Wang, Hongyi Jiang, Shuang Peng, Xiaokun Zhao

**Affiliations:** 1 Department of Urology, The Second Xiangya Hospital, Central South University, Changsha, Hunan 410011, P.R. China

**Keywords:** clear cell renal cell carcinoma, LINC00511, microRNA-625, cyclin D1

## Abstract

The expression pattern and detailed roles of long noncoding RNA LINC00511 in clear cell renal cell carcinoma (ccRCC) remain unknown. We measured LINC00511 expression in ccRCC. We clarified the clinical characteristics associated with LINC00511 in ccRCC. We examined the biological roles of LINC00511 in the progression of ccRCC, and we identified the potential mechanisms involved. LINC00511 was upregulated in ccRCC tissues and cell lines. High LINC00511 expression significantly correlated with TNM classification, lymph node metastasis, and short overall survival among patients with ccRCC. Additionally, LINC00511 knockdown restricted ccRCC cell proliferation, colony formation, and metastasis *in vitro*; accelerated cell cycle arrest at G0–G1 and apoptosis *in vitro*; and decreased tumor growth *in vivo*. Investigation of the mechanism revealed that LINC00511 directly interacted with microRNA-625 (miR-625), and the inhibitory effects of the LINC00511 knockdown on malignant characteristics were neutralized by miR-625 silencing. Furthermore, cyclin D1 (*CCND1*) was identified as a direct target of miR-625 in ccRCC cells. The tumor-suppressive activity of miR-625 upregulation on ccRCC cells was reversed by CCND1 reintroduction. In conclusion, LINC00511 serves as a competing endogenous RNA that regulates CCND1 expression by sponging miR-625 in ccRCC. Hence, the LINC00511/miR-625/CCND1 pathway might be a promising therapeutic target in ccRCC.

## INTRODUCTION

Renal cell carcinoma (RCC), originating in the renal cortex, is one of the most common types of urological tumors [1]. RCC is characterized by a lack of obvious early clinical symptoms, diversity of clinical manifestations, and resistance to chemoradiotherapy [2]. Approximately ~295,000 novel RCC cases are diagnosed, and ~134,000 deaths occur because of RCC worldwide each year [3]. Clear cell RCC (ccRCC) is the most prevalent pathological subtype and accounts for approximately 70% of all diagnosed RCC cases [4]. Currently, nephrectomy remains the standard curative treatment for patients with localized ccRCC [5]. Despite significant progress in the development of treatments, the long-term prognosis of patients with metastatic ccRCC remains poor, with a median survival period of only 1.5 years [6]. Multiple risk factors, including dietary habits, physical activity, and occupational exposure to specific carcinogens, are known to be closely linked with ccRCC genesis and progression [7]. Nevertheless, the detailed mechanisms underlying the malignant progression of ccRCC remain unclear. Therefore, an in-depth understanding of the molecular interactions involved in the initiation and progression of ccRCC may help to develop effective therapeutic methods for patients with this malignant tumor.

Long noncoding RNAs (lncRNAs) are a series of non-protein-coding RNA molecules over 200 nucleotides long [8] that are involved in various biological events, including genomic imprinting, epigenetic regulation, alternative splicing, cell differentiation, and tumorigenesis [9, 10]. Recently, a variety of lncRNAs were found to be aberrantly expressed during the initiation and progression of ccRCC. For example, XIST [11], OTUD6B-AS1 [12], and DHRS4-AS1 [13] are downregulated in ccRCC. On the contrary, ITGB1 [14], ZFAS1 [15], and AFAP1-AS1 [16] are overexpressed in ccRCC. LncRNAs exert either tumor-suppressive or oncogenic effects during carcinogenesis, including cancer progression. These actions are mediated by different mechanisms, including RNA decoys; alternative splicing; and epigenetic, transcriptional, and post-transcriptional modifications [17, 18]. Therefore, identifying ccRCC-associated lncRNAs and elucidating their involvement in cancer progression might contribute to the development of molecular targets for the diagnosis and treatment of ccRCC.

MicroRNAs (miRNAs) belong to a series of highly conserved, noncoding short RNA molecules of approximately 18–25 nucleotides [19]. miRNAs effectively downregulate gene expression by imperfectly or perfectly binding to the 3′-untranslated regions (3′-UTRs) of their target genes. This results in mRNA degradation and/or translation repression [20]. Over 1500 mature miRNA genes have been identified in the human genome. These miRNAs participate in the regulation of various physiological and pathological phenomena, such as cell proliferation, cell cycle, apoptosis, differentiation, metastasis, and angiogenesis [21–23]. Numerous studies have demonstrated that miRNAs are dysregulated in nearly all cancer types, including ccRCC [24, 25]. miRNAs in ccRCC have both tumor suppressive and oncogenic potential, depending on the characteristics of their target genes [26]. For example, miR-1274a is overexpressed in ccRCC. This overexpression promotes cell proliferation and inhibits cell apoptosis by directly targeting the tumor suppressor BMPR1B [27]. Therefore, functional miRNAs may be important for ccRCC prognosis and as therapeutic targets. An in-depth investigation of miRNA functions in ccRCC may facilitate the identification of potential targets for anticancer therapies.

LINC00511 is an lncRNA and has been previously reported to be implicated in the carcinogenesis and progression of multiple human cancer types [28–38]. However, the expression pattern and detailed roles of LINC00511 in ccRCC are still unknown. Therefore, the aims of this study were to evaluate LINC00511 expression in ccRCC, clarify the clinical characteristics associated with LINC00511 in ccRCC, examine the biological roles of LINC00511 in the progression of ccRCC, and reveal the potential mechanisms involved.

## RESULTS

### LINC00511 is upregulated in ccRCC and correlates with poor clinical outcomes

**Figure 1:**
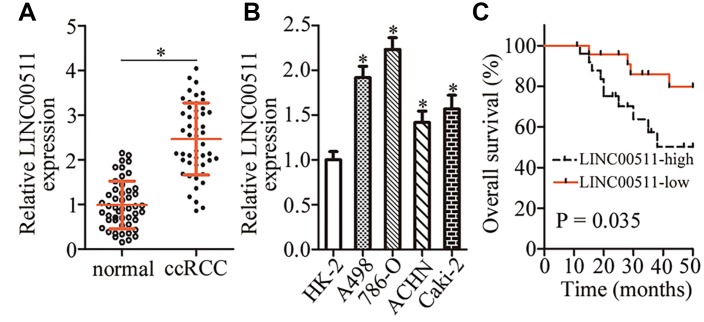
**LINC00511 is upregulated in ccRCC tissues and cell lines.** (**A**) RT-qPCR analysis was conducted to quantitate the expression of LINC00511 in 49 pairs of ccRCC samples and matched adjacent normal renal tissue samples. *P < 0.05 vs. normal renal tissues. (**B**) Relative LINC00511 expression in four ccRCC cell lines (A498, 786-O, ACHN, and Caki-2) and one normal human renal cell line (HK-2) was quantified by RT-qPCR. *P < 0.05 vs. HK-2 cells. (**C**) The overall survival of patients with ccRCC harboring low or high LINC00511 levels was studied by Kaplan–Meier survival analysis, and the curves were compared by the logrank test. P = 0.035.

To analyze the expression pattern of LINC00511 in ccRCC, we first measured LINC00511 expression in 49 pairs of ccRCC samples and matched adjacent normal renal tissue samples. RT-qPCR analysis showed that LINC00511 was upregulated in ccRCC tissue samples compared with the adjacent normal renal tissues (Figure 1A, P < 0.05). In addition, we found that the expression of LINC00511 was considerably higher in all four ccRCC cell lines than in normal human renal (HK-2) cells (Figure 1B, P < 0.05).

We next investigated the clinical characteristics associated with LINC00511 in ccRCC. All patients with ccRCC were subdivided into two groups: the LINC00511 high expression group (n = 25) and LINC00511 low expression group (n = 24). This classification was based on the median value of LINC00511 expression among the ccRCC tissue samples. Statistical analysis revealed that an increased LINC00511 level was strongly related to the TNM classification (P = 0.015) and lymph node metastasis (P = 0.027) among the patients with ccRCC (Table 1). Furthermore, the patients with ccRCC harboring higher LINC00511 levels showed significantly shorter overall survival than did the ccRCC patients with lower LINC00511 expression (Figure 1C, P = 0.035). These results suggested that LINC00511 is overexpressed in ccRCC and may be closely related to ccRCC formation and progression.

**Table 1:** The correlation between LINC00511 expression and clinical characteristics of patients with ccRCC.

**Characteristics**	**Cases**	**LINC00511 expression**		**P value**
		**High**	**Low**	
**Gender**				0.680
Male	21	10	11	
Female	28	15	13	
**Age**				0.470
<65 years	35	19	16	
≥ 65 years	14	6	8	
**Tumor size**				0.458
<4 cm	28	13	15	
≥ 4 cm	21	12	9	
**Grade**				0.482
Grade 1+2	22	10	12	
Grade 3+4	27	15	12	
**TNM classification**				0.015^a^
I+II	26	9	17	
III+IV	23	16	7	
**Lymph node metastasis**				0.027^a^
Negative	29	11	18	
Positive	20	14	6	

^a^P < 0.05 (chi-square test).

### LINC00511 knockdown inhibits the growth and metastasis of ccRCC cells *in vitro*

**Figure 2:**
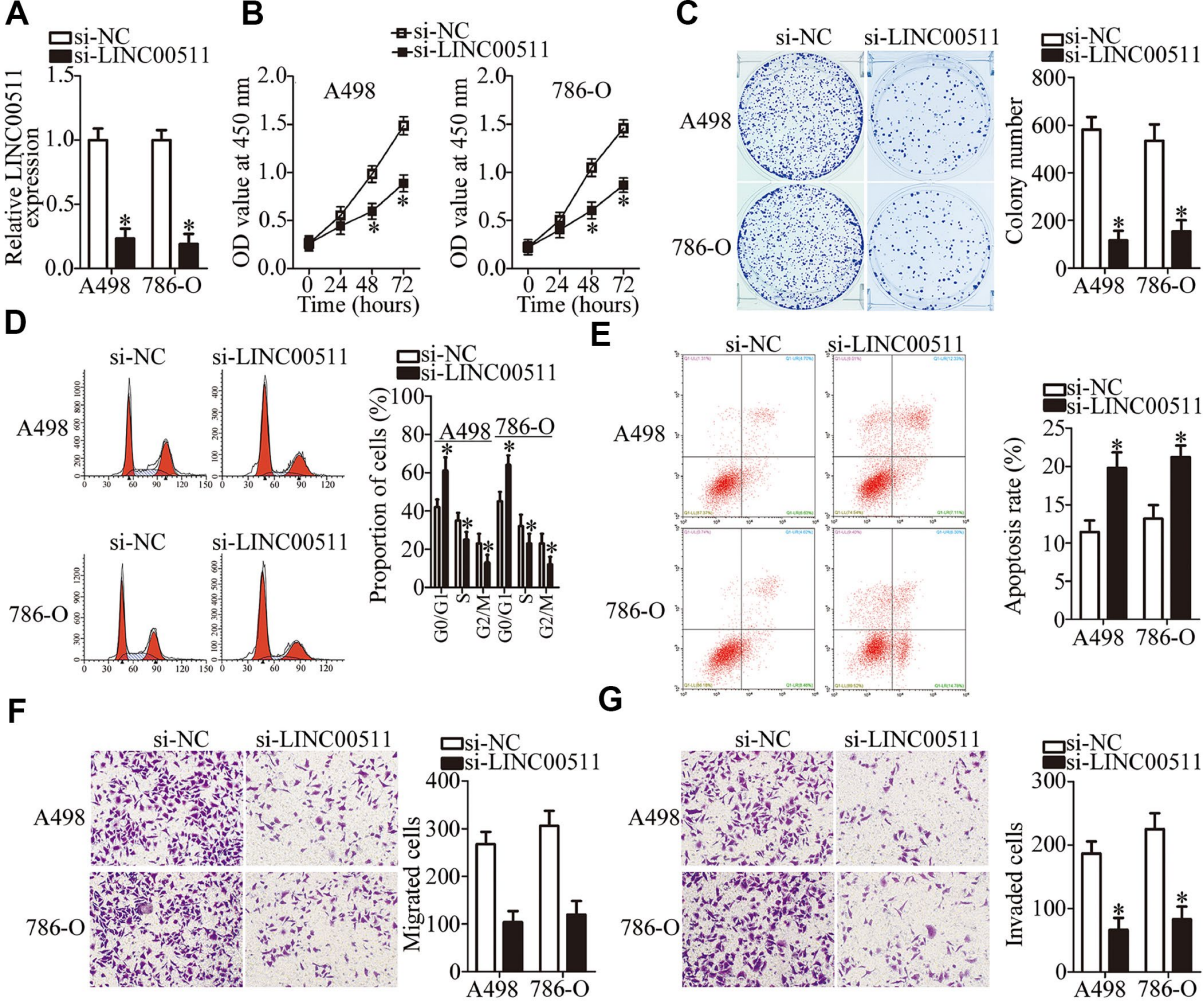
**LINC00511 knockdown inhibits ccRCC proliferation and induces apoptosis of A498 and 786-O cells.** (**A**) A498 and 786-O cells were transfected with either si-LINC00511 or si-NC. After transfection, LINC00511 expression was determined by RT-qPCR. *P < 0.05 vs. the si-NC group. (**B**) CCK-8 assays were performed to measure the proliferative ability of A498 and 786-O cells treated with either si-LINC00511 or si-NC. *P < 0.05 vs. group “si-NC.” (**C**) Colony formation assays of A498 and 786-O cells transfected with either si-LINC00511 or si-NC. Representative images for each treatment are shown. *P < 0.05 vs. the si-NC group. (**D**, **E**) Cell cycle and apoptosis assays were performed to determine the cell cycle status and apoptotic rate of A498 and 786-O cells after transfection with either si-LINC00511 or si-NC. *P < 0.05 vs. group si-NC. (**F**, **G**) Si-LINC00511 or si-NC was introduced into A498 and 786-O cells. Migration and invasion abilities were assessed by Transwell migration and invasion assays. *P < 0.05 vs. the si-NC group.

To characterize the detailed roles of LINC00511 in ccRCC progression, A498 and 786-O cells were selected for a loss-of-function analysis. These cells were transfected with si-LINC00511 and si-NC. LINC00511 was efficiently silenced in A498 and 786-O cells transfected with si-LINC00511 (Figure 2A, P < 0.05). The functional effect of the LINC00511 knockdown on ccRCC cell proliferation was examined by CCK-8 assays. The data indicated that the silenced LINC00511 expression significantly impeded the proliferation of A498 and 786-O cells (Figure 2B, P < 0.05). Next, colony formation assays were conducted to confirm the ccRCC cell proliferation suppression caused by the LINC00511 knockdown. In contrast to the cells transfected with si-NC, si-LINC00511–transfected A498 and 786-O cells yielded fewer and smaller colonies (Figure 2C, P < 0.05).

Alterations in cell proliferation mostly correlate with cell cycle progression and apoptosis, indicating that cell cycle arrest and apoptosis lead to growth inhibition. A clear increase in the proportion of G0–G1 transition cells and a notable decrease in the proportion of S phase cells were observed among A498 and 786-O cells transfected with si-LINC00511 as compared to the cells transfected with si-NC (Figure 2D, P < 0.05). This suggests that depletion of LINC00511 caused G0–G1 arrest. The effect of LINC00511 on the apoptosis of ccRCC cells was also examined. This experiment revealed that the knockdown of LINC00511 led to an obvious increase in the apoptotic rate of A498 and 786-O cells (Figure 2E, P < 0.05). These results indicate that the knockdown of LINC00511 inhibits ccRCC cell proliferation by inducing G0–G1 phase arrest and facilitating apoptosis.

Because the LINC00511 expression levels correlate with lymph node metastasis, we hypothesized that LINC00511 is involved in the regulation of ccRCC metastasis. To test this hypothesis, Transwell migration and invasion assays were conducted to evaluate the migration and invasion of A498 and 786-O cells upon LINC00511 downregulation. The knockdown of LINC00511 reduced the migratory ability of A498 and 786-O cells relative to that in cells transfected with si-NC (Figure 2F, P < 0.05). Similarly, transfection with si-LINC00511 significantly decreased the number of invaded A498 and 786-O cells in comparison with that in the si-NC groups (Figure 2G, P < 0.05). Collectively, these results implied that the LINC00511 knockdown exerts an inhibitory action on ccRCC growth and metastasis in vitro.

### LINC00511 functions as competitive endogenous RNA (ceRNA) for miR-625 in ccRCC

**Figure 3:**
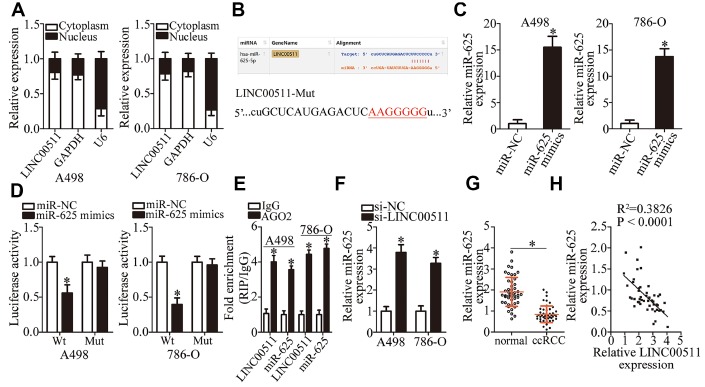
**LINC00511 directly sponges miR-625 in ccRCC cells.** (**A**) Nuclear/cytoplasmic fractionation analysis of LINC00511 expression in ccRCC cells. (**B**) The binding site in LINC00511 for miR-625, as revealed by bioinformatics analysis. The mutant binding sequences in LINC00511 are also shown. (**C**) A498 and 786-O cells were transfected with the miR-625 mimics or miR-NC. The transfected cells were collected after 48 h incubation and then subjected to RT-qPCR analysis to determine transfection efficiency. *P < 0.05 vs. the miR-NC group. (**D**) Either LINC00511-Wt or LINC00511-Mut was cotransfected into A498 and 786-O cells with either the miR-625 mimics or miR-NC. After 48 h transfection, the detection of luciferase activity was conducted via a Dual-Luciferase Reporter System. *P < 0.05 vs. the miR-NC group. (**E**) A RIP assay was conducted to assess the direct interaction between LINC00511 and miR-625. LINC00511 and miR-625 were both immunoprecipitated by the anti-AGO2 antibody from the lysates of A498 and 786-O cells. *P < 0.05 vs. the IgG group. (**F**) miR-625 expression was quantified in the presence of either si-LINC00511 or si-NC by RT-qPCR. *P < 0.05 vs. the si-NC group. (**G**) The expression levels of miR-625 in 49 pairs of ccRCC samples and matched adjacent normal renal tissue samples were measured via RT-qPCR. *P < 0.05 vs. normal renal tissues. *P < 0.05 vs. normal renal tissues. (**H**) An inverse expression correlation between LINC00511 and miR-625 in ccRCC tissue samples was identified by Spearman’s correlation analysis. R^2^ = 0.3826, P < 0.0001.

Increasing evidence suggests that lncRNAs can serve as a ceRNA by sponging certain miRNAs [39–41]. To elucidate the mechanisms behind the activity of LINC00511 in ccRCC, the nuclear/cytoplasmic fractionation assay was carried out. This assay indicated that LINC00511 was primarily located in the cytoplasm of ccRCC cells (Figure 3A), suggesting that LINC00511 may function as an miRNA sponge in ccRCC. First, using the bioinformatics tool (starBase 3.0), we determined that LINC00511 contains a putative binding site for miR-625 (Figure 3B). To confirm this prediction, the luciferase reporter assay was performed on A498 and 786-O cells after cotransfection with either LINC00511-Wt or LINC00511-Mut and either the miR-625 mimics or miR-NC. The results showed that transfection with the miR-625 mimics, which resulted in obvious upregulation of miR-625 (Figure 3C, P < 0.05), decreased the luciferase activity of LINC00511-Wt (P < 0.05). By contrast, the luciferase activity of LINC00511-Mut stayed unaltered in A498 and 786-O cells after miR-625 upregulation (Figure 3D). The RIP assay was then performed to characterize the direct interaction between LINC00511 and miR-625 in ccRCC cells. The results revealed that LINC00511 and miR-625 were both immunoprecipitated by the anti-AGO2 antibody from the lysates of A498 and 786-O cells. These results suggest that miR-625 is an LINC00511-targeted miRNA (Figure 3E, P < 0.05).

We next applied RT-qPCR analysis to determine the expression levels of miR-625 in A498 and 786-O cells in response to LINC00511 silencing. miR-625 expression was obviously enhanced in A498 and 786-O cells after inhibition of LINC00511 expression (Figure 3F, P < 0.05). Furthermore, miR-625 was found to be downregulated in ccRCC tissue samples relative to adjacent normal renal tissues (Figure 3G, P < 0.05). Moreover, an inverse expression correlation between LINC00511 and miR-625 was identified among the same ccRCC tissue samples, as evidenced by Spearman’s correlation analysis (Figure 3H; R^2^ = 0.3826, P < 0.0001). Taken together, these results provided sufficient evidence to demonstrate that LINC00511 directly sponges miR-625 in ccRCC cells.

### miR-625 works as a tumor-suppressive miRNA during ccRCC progression

**Figure 4:**
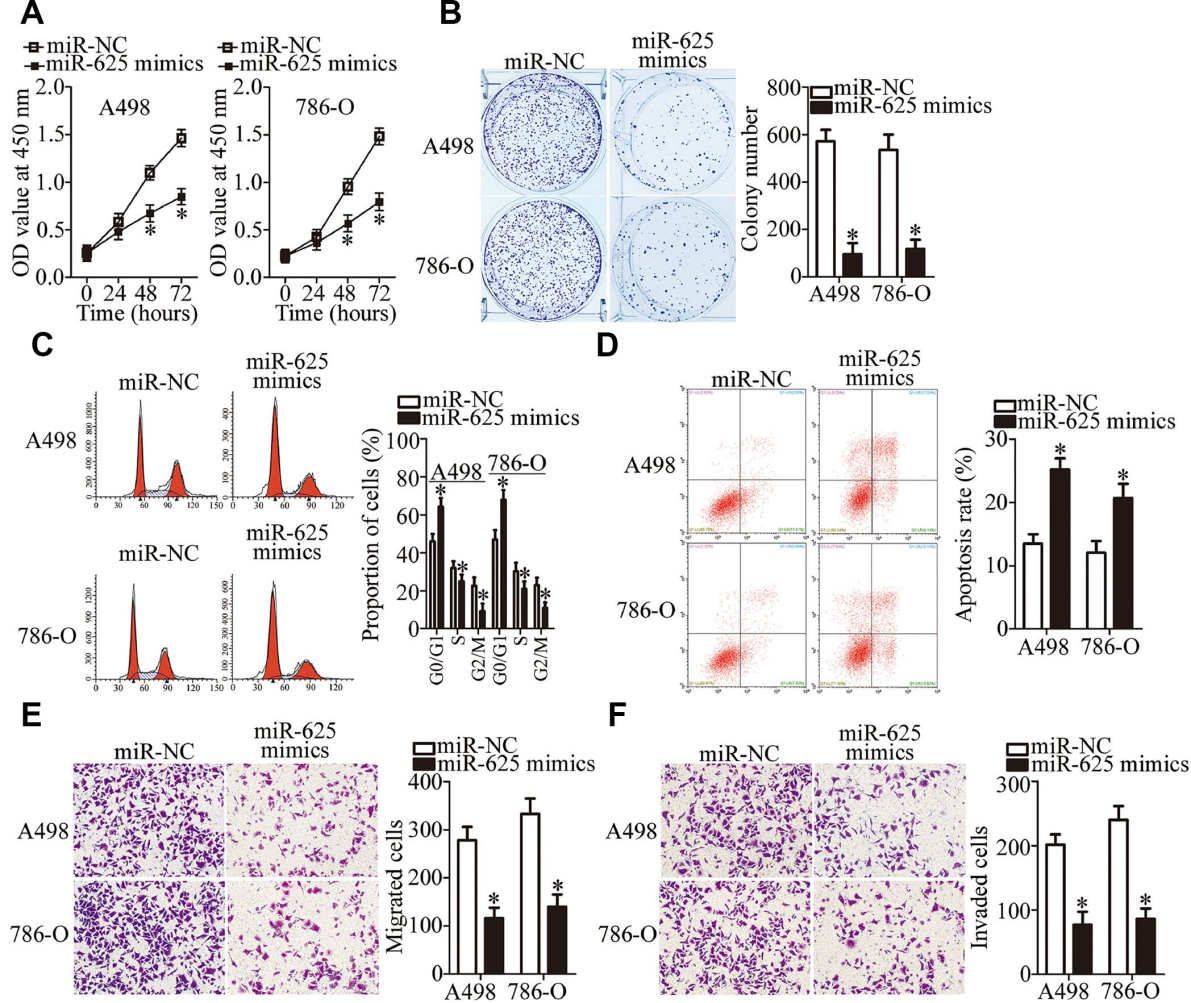
**miR-625 upregulation inhibits the growth and metastasis of ccRCC cells in vitro.** (**A**, **B**) A498 and 786-O cells transfected with the miR-625 mimics or miR-NC were collected. These cells were subjected to CCK-8 and colony formation assays to determine cell proliferation and colony formation capacity, respectively. *P < 0.05 vs. the miR-NC group. (**C**, **D**) Flow-cytometric determination of the cell cycle distribution and apoptotic rate of miR-625–overexpressing A498 and 786-O cells. *P < 0.05 vs. group miR-NC. (**E**, **F**) The impact of miR-625 overexpression on A498 and 786-O cell migration and invasion was determined in Transwell migration and invasion assays, respectively. Representative images and quantification are presented. *P < 0.05 vs. group miR-NC.

Because miR-625 was found to be sponged by LINC00511 in ccRCC, we next evaluated the biological functions of miR-625 in ccRCC cells. Either the miR-625 mimics or miR-NC was introduced into A498 and 786-O cells. After transfection, a series of functional experiments was conducted. The results showed that forced miR-625 expression attenuated A498 and 786-O cell proliferation (Figure 4A, P < 0.05) and colony formation abilities (Figure 4B, P < 0.05). Forced miR-625 expression also promoted G0–G1 cell cycle arrest (Figure 4C, P < 0.05) and cell apoptosis (Figure 4D, P < 0.05), and restricted migration (Figure 4E, P < 0.05) and invasion (Figure 4F, P < 0.05) *in vitro*. These results indicated a tumor-suppressive influence of miR-625 on the malignancy of ccRCC.

### *CCND1* is a direct target gene of miR-625 in ccRCC cells

**Figure 5:**
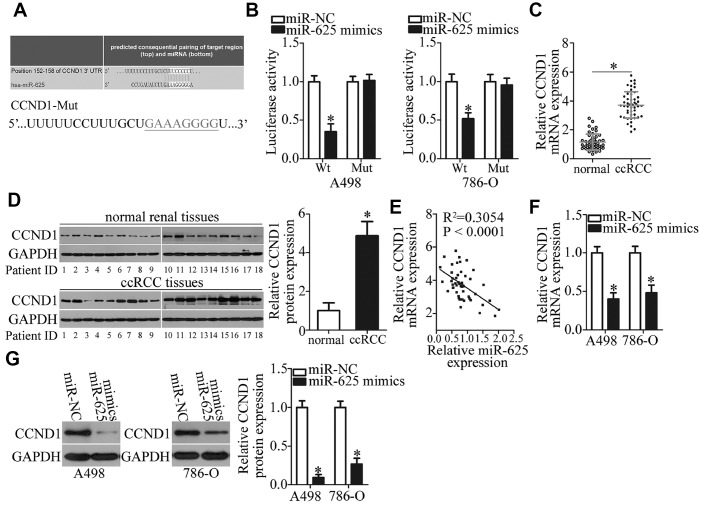
***CCND1* is a direct target gene of miR-625 in ccRCC cells.** (**A**) A putative binding site for miR-625 in the 3′-UTR of *CCND1* was predicted by starBase 3.0, TargetScan, microRNA.org, and miRDB. The mutant binding sequences for miR-625 in the 3′-UTR of CCND1 are also shown. (**B**) Luciferase activity was measured in A498 and 786-O cells cotransfected with a reporter plasmid carrying either the wild-type or mutant *CCND1* 3′-UTR and either the miR-625 mimics or miR-NC. *P < 0.05 vs. the miR-NC group. (**C**) RT-qPCR was performed to analyze CCND1 mRNA expression in ccRCC samples and in matched adjacent normal renal tissues. *P < 0.05 vs. normal renal tissue samples. (**D**) The protein levels of CCND1 were measured in the ccRCC samples and in matched adjacent normal renal tissue samples by western blotting. *P < 0.05 vs. normal renal tissues. (**E**) The association between miR-625 and *CCND1* mRNA levels in ccRCC tissue samples was evaluated by Spearman’s correlation analysis. R^2^ = 0.3054, P < 0.0001. (**F**, **G**) CCND1 mRNA and protein levels in A498 and 786-O cells transfected with either the miR-625 mimics or miR-NC were investigated by RT-qPCR and western blotting, respectively. *P < 0.05 vs. the miR-NC group.

To determine the mechanism by which miR-625 performs tumor-suppressive functions in ccRCC, bioinformatics analysis was performed to search for the putative targets of miR-625. *CCND1* was found to be a major target of miR-625 according to all four bioinformatics databases (Figure 5A). Therefore, *CCND1* was chosen for validation, because CCND1 is frequently reported to participate in the formation and progression of multiple human cancer types [42, 43]. Based on this prediction, luciferase reporter plasmids were constructed and used in luciferase reporter assays. The results revealed that luciferase activity was considerably decreased in A498 and 786-O cells cotransfected with the miR-625 mimics and a reporter plasmid harboring the wild-type miR-625–binding site (P < 0.05). Cells cotransfection of the miR-625 mimics and mutant *CCND1* 3′-UTR failed to increase or decrease luciferase activity (Figure 5B).

To further illustrate the relation between miR-625 and CCND1 in ccRCC, we measured CCND1 expression in the 49 pairs of ccRCC samples and matched adjacent normal renal tissue samples by RT-qPCR. The CCND1 mRNA level was dramatically higher in ccRCC tissue samples than in adjacent normal renal tissues (Figure 5C, P < 0.05). In addition, CCND1 protein expression was excessive in ccRCC tissue samples as compared to that in adjacent normal renal tissues (Figure 5D, P < 0.05). While comparing miR-625 and CCND1 expression among these ccRCC tissue samples, we identified a negative correlation between miR-625 and *CCND1* mRNA levels among these 49 ccRCC tissue samples (Figure 5E; R^2^ = 0.3054, P < 0.0001). Furthermore, RT-qPCR and western blotting proved that the ectopic miR-625 expression significantly downregulated CCND1 in A498 and 786-O cells at both the mRNA (Figure 5F, P < 0.05) and protein (Figure 5G, P < 0.05) levels. Taken together, these results suggested that CCND1 is a direct target of miR-625 in ccRCC cells.

### CCND1 restoration abrogates the tumor-suppressive roles of miR-625 overexpression in ccRCC cells

**Figure 6:**
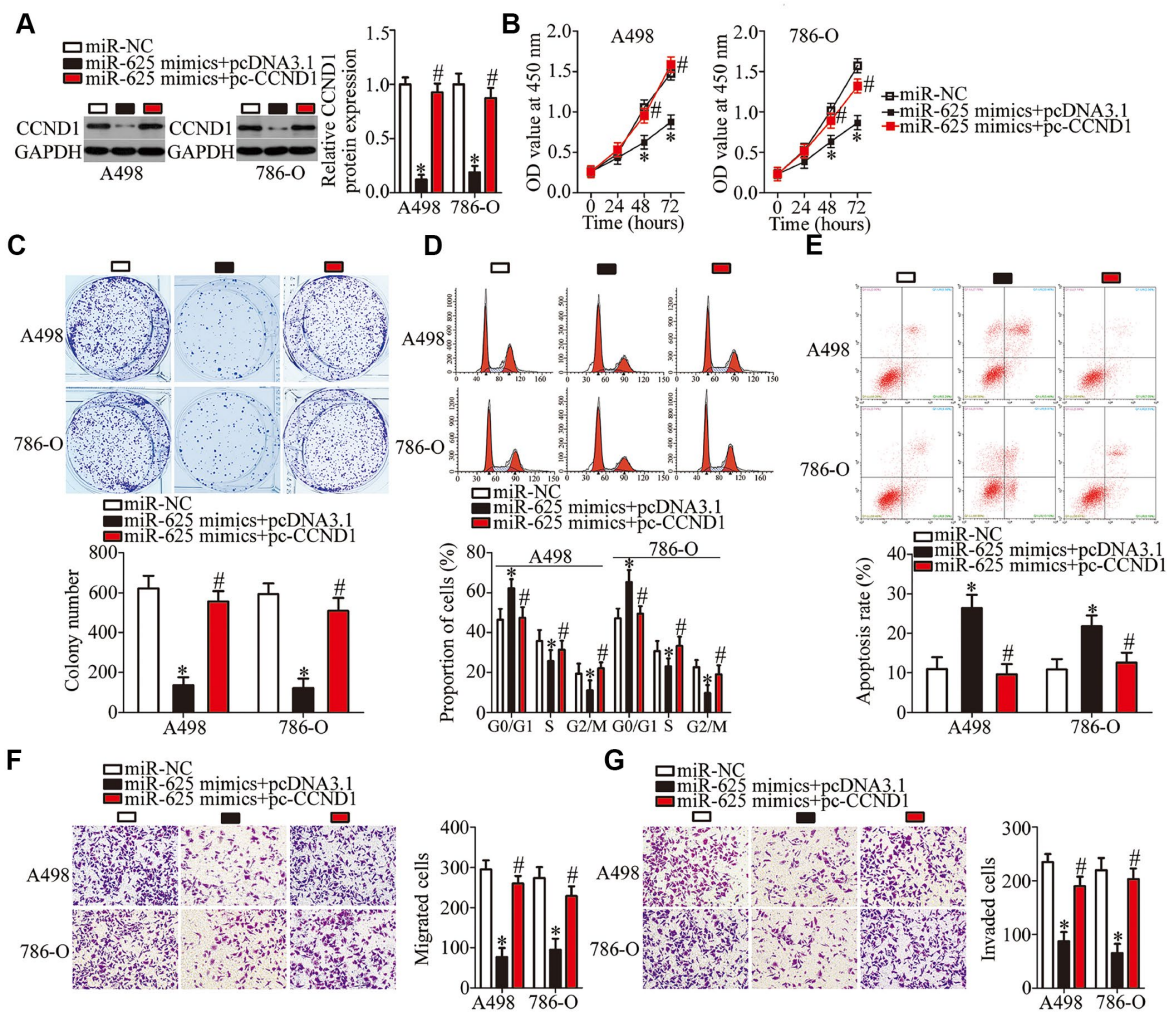
**CCND1 reintroduction partially reverses the effects of miR-625 overexpression on A498 and 786-O cells.** A498 and 786-O cells were transfected with the miR-625 mimics along with pcDNA3.1 or pc-CCND1 without 3′-UTR and were subjected to the following assays. (**A**) CCND1 protein expression was quantitated by western blotting. *P < 0.05 vs. group miR-NC. ^#^P < 0.05 vs. group miR-625 mimics+pcDNA3.1. (**B**–**G**) The CCK-8 assay, colony formation assay, cell cycle assay, cell apoptosis assay, and Transwell migration and invasion assays were conducted to determine the proliferation, colony formation, cell cycle status, apoptosis, migration, and invasion of the previously described cells, respectively. *P < 0.05 vs. the miR-NC group. ^#^P < 0.05 vs. group miR-625 mimics+pcDNA3.1.

A series of rescue experiments was conducted to test whether CCND1 mediates the tumor-suppressive actions of miR-625 overexpression in ccRCC cells. The miR-625 mimics, along with pcDNA3.1 or pc-CCND1 without the 3′-UTR, were transfected into A498 and 786-O cells. Downregulation of the *CCND1* protein under the influence of miR-625 overexpression was reversed in A498 and 786-O cells by cotransfection with pc-CCND1 (Figure 6A, P < 0.05). Furthermore, the results of functional assays showed that restoration of CCND1 expression partially reversed the impact of miR-625 overexpression on the proliferation (Figure 6B, P < 0.05), colony formation (Figure 6C, P < 0.05), cell cycle status (Figure 6D, P < 0.05), apoptosis (Figure 6E, P < 0.05), migration (Figure 6F, P < 0.05), and invasiveness (Figure 6G, P < 0.05) of A498 and 786-O cells. These results indicated that miR-625 inhibits the initiation and progression of ccRCC, at least partly, by reducing CCND1 expression.

### LINC00511 exerts oncogenic actions in ccRCC cells via the miR-625–CCND1 axis

**Figure 7:**
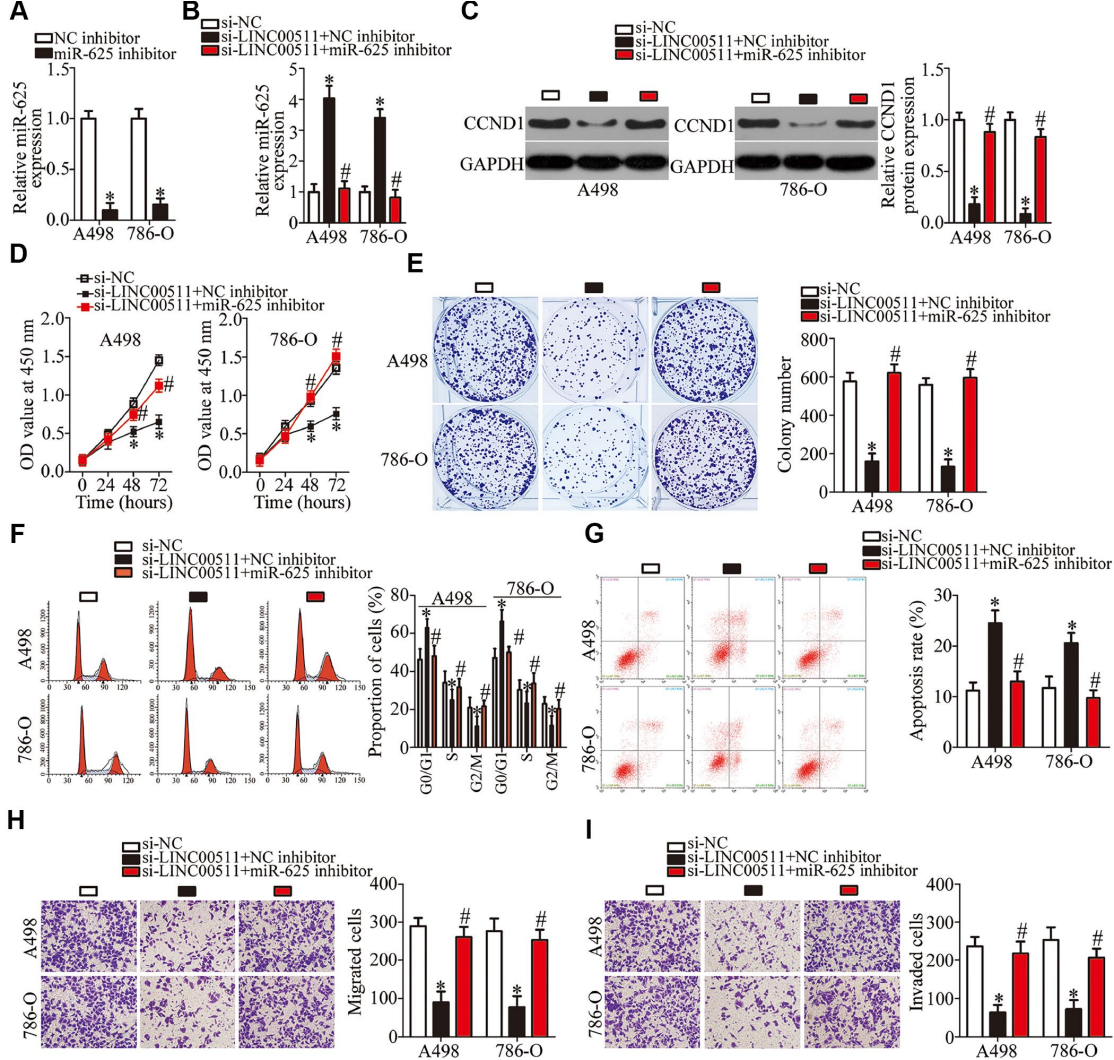
**Silencing of miR-625 expression neutralizes the actions of the LINC00511 knockdown on ccRCC cells.** LINC00511-deficient A498 and 786-O cells were treated with either the miR-625 inhibitor or NC inhibitor. Transfected cells were used in the subsequent functional experiments. (**A**) The expression levels of miR-625 in A498 and 786-O cells after miR-625 inhibitor or NC inhibitor transfection were measured via RT-qPCR. *P < 0.05 vs. the NC inhibitor group. (**B**, **C**) miR-625 and CCND1 protein amounts in the above-mentioned cells were determined by RT-qPCR and western blotting, respectively. *P < 0.05 vs. the si-NC group. ^#^P < 0.05 vs. group si-LINC00511+NC inhibitor. (**D**–**I**) The proliferation, colony formation, cell cycle status, apoptosis, migration, and invasiveness of A498 and 786-O cells cotransfected with si-LINC00511 and either the miR-625 inhibitor or NC inhibitor were analyzed by the CCK-8 assay, colony formation assay, cell cycle assay, cell apoptosis assay, and Transwell migration and invasion assays, respectively. *P < 0.05 vs. the si-NC group. ^#^P < 0.05 vs. group si-LINC00511+NC inhibitor.

To further clarify whether the miR-625–CCND1 axis is responsible for the impact of LINC00511 on ccRCC progression, rescue experiments were carried out by introducing the miR-625 inhibitor into LINC00511-deficient A498 and 786-O cells. The efficiency of miR-625 inhibitor transfection was validated via RT-qPCR (Figure 7A, P < 0.05). The increase in miR-625 abundance (Figure 7B, P < 0.05) and a decrease in the CCND1 protein amount (Figure 7C, P < 0.05) in A498 and 786-O cells, as a consequence of LINC00511 silencing, were reversed by cotransfection with the miR-625 inhibitor. The recovery of miR-625 expression abrogated the effects of the LINC00511 knockdown on A498 and 786-O cell proliferation (Figure 7D, P < 0.05), colony formation (Figure 7E, P < 0.05), apoptosis (Figure 7F, P < 0.05), cell cycle (Figure 7G, P < 0.05), migration (Figure 7H, P < 0.05), and invasion (Figure 7I, P < 0.05). Above all, the miR-625–CCND1 axis is the functional mediator of LINC00511 in ccRCC cells.

### The knockdown of LINC00511 represses ccRCC tumor growth *in vivo*

**Figure 8:**
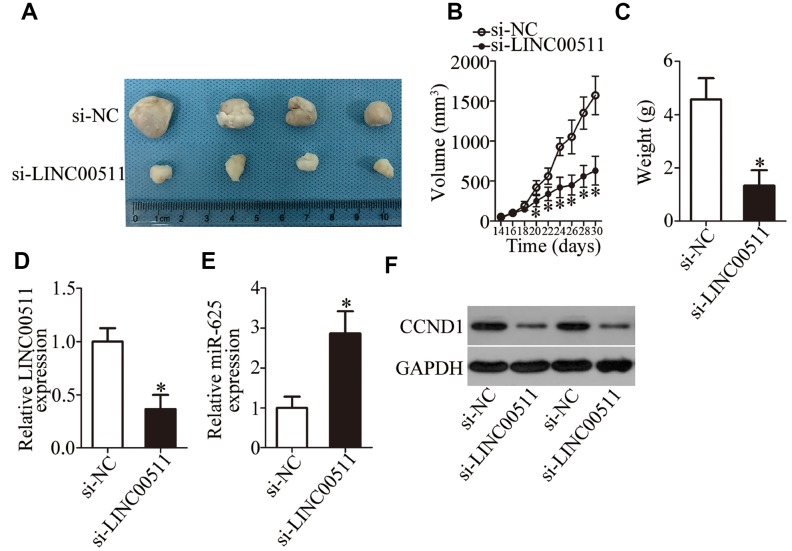
**Reduction in LINC00511 expression inhibits ccRCC tumor growth in vivo.** (**A**) Representative images of xenograft tumors derived from si-LINC00511–transfected or si-NC–transfected A498 cells. (**B**) Tumor volumes in the BALB/c nude mice of si-LINC00511 and si-NC groups were calculated every 2 days and growth curves were generated. *P < 0.05 vs. group si-NC. (**C**) The tumor xenografts in the si-LINC00511 and si-NC groups of BALB/c nude mice were excised and weighed. *P < 0.05 vs. group si-NC. (**D**, **E**) Expression levels of LINC00511 and miR-625 in xenograft tumors were quantified by RT-qPCR. LINC00511 expression was found to be decreased, while miR-625 expression was high in the xenografts of the si-LINC00511 group compared to the si-NC group. *P < 0.05 vs. the si-NC group. (**F**) Protein expression of CCND1 in the xenografts of si-LINC00511 and si-NC groups was determined by western blotting.

To investigate the precise role of LINC00511 *in vivo*, nude mice were subcutaneously inoculated with 786-O cells transfected with either si-LINC00511 or si-NC. The results showed that the tumor volume was significantly lower in the si-LINC00511 group compared to the si-NC group (Figure 8A and 8B, P < 0.05). BALB/c nude mice were euthanized 30 days after injection. Xenografts were excised and weighed. LINC00511-deficient xenografts manifested obvious tumor weight suppression as compared to the si-NC group (Figure 8C, P < 0.05). Additionally, RT-qPCR analysis was carried out to detect LINC00511 and miR-625 expression in the tumor xenografts. LINC00511 expression was found to be decreased (Figure 8D, P < 0.05), while miR-625 expression was increased (Figure 8E, P < 0.05) in the tumor xenografts derived from si-LINC00511–transfected 786-O cells. Furthermore, western blot analysis revealed that the protein level of CCND1 was obviously lower in the si-LINC00511 group than in the si-NC group (Figure 8F). Taken together, these results indicated that the LINC00511 knockdown inhibits ccRCC tumorigenicity *in vivo* by regulating the miR-625–CCND1 axis.

## DISCUSSION

An increasing number of studies have shown that numerous lncRNAs are deregulated in ccRCC. Additionally, these studies have shown that the deregulation is involved in ccRCC pathogenesis and progression through their participation in a variety of critical biological processes [44–46]. Strategies for inhibiting the aggressiveness of various human cancer types by means of lncRNAs have been proposed in recent studies [47–49]. Therefore, further studies on the expression and biological functions of lncRNAs in ccRCC may facilitate the development of novel therapeutic techniques for patients carrying this malignant tumor. In this study, we attempted to unveil the expression profile of LINC00511 in ccRCC and evaluate its clinical significance among patients with ccRCC. The detailed involvement of LINC00511 in the malignancy of ccRCC and the underlying molecular mechanisms were explored. Our findings uncovered crucial participation of the LINC00511–miR-625–CCND1 axis in the aggressiveness of ccRCC.

LINC00511 is dysregulated in multiple types of human cancer. For instance, LINC00511 is overexpressed in the tissues and cell lines of cervical cancer. High LINC00511 expression strongly correlates with the tumor stage, tumor size, and lymph node metastasis in cervical cancer [28]. LINC00511 is upregulated in hepatocellular carcinoma. Its upregulation is related to lymph node metastasis, vascular invasion, and clinical stage [29]. Patients with hepatocellular carcinoma featuring high LINC00511 expression have worse overall survival rates [29]. Moreover, expression of LINC00511 is reported to be an independent prognostic factor of overall survival among patients with hepatocellular carcinoma [29]. LINC00511 is also overexpressed in non–small cell lung cancer [30], pancreatic ductal adenocarcinoma [31], tongue squamous cell carcinoma [32], breast cancer [33, 34], osteosarcoma [35], glioma [36], ovarian cancer [37], and thyroid carcinoma [38]. Nonetheless, few studies have illustrated the expression status of LINC00511 in ccRCC. Herein, we found that LINC00511 is significantly upregulated in ccRCC tissue samples and cell lines. The increased LINC00511 expression was found to be strongly associated with the TNM stage and lymph node metastasis among patients with ccRCC. The patients with ccRCC harboring high LINC00511 levels presented significantly shorter overall survival than the patients with ccRCC showing low LINC00511 expression.

Aberrations of LINC00511 expression perform important functions in tumorigenesis and tumor progression. For instance, depletion of the *LINC00511* gene inhibits cell resistance to PTX, cell viability, cell proliferation, and metastasis, and promotes apoptosis in cervical cancer [28]. LINC00511 silencing attenuates the proliferation and motility of hepatocellular carcinoma cells [29]. In pancreatic ductal adenocarcinoma, knockdown of LINC00511 suppresses cell proliferation, migration, and invasion decreases endothelial tube formation [31]. In breast cancer, reduction in LINC00511 expression restricts breast cancer cell proliferation, sphere-formation ability, stem factors in vitro; hinders tumor growth in vivo; and increases paclitaxel cell cytotoxicity [33, 34]. LINC00511 also acts as an oncogene in non–small cell lung cancer [30], tongue squamous cell carcinoma [32], osteosarcoma [35], glioma [36], ovarian cancer [37], and thyroid carcinoma [38]. In the present study, functional experiments revealed that silencing of LINC00511 expression restricts ccRCC cell proliferation and the colony formation ability of these cells; induces G0–G1 cell cycle arrest; promotes apoptosis; and reduces cell migration and invasion *in vitro* and inhibits tumor growth *in vivo*.

Identification of the molecular events related to the oncogenic actions of LINC00511 on ccRCC may be helpful for exploring effective therapeutic strategies. Accordingly, the mechanisms behind the oncogenic actions of LINC00511 in ccRCC were elucidated here at the molecular level. We revealed that LINC00511 functions as a ceRNA for miR-625 in ccRCC. miR-625 is under-expressed in multiple human cancer types, including colorectal cancer [50], breast cancer [51], laryngeal squamous cell carcinoma [52], gastric cancer [53], esophageal cancer [54], and hepatocellular carcinoma [55]. Functionally, miR-625 exerts tumor-suppressive actions on the aforementioned human cancer types and is implicated in the regulation of various malignant characteristics [50–55]. Here, our solid data indicate that miR-625 is weakly expressed in ccRCC. Exogenous miR-625 expression suppressed the malignancy of ccRCC cells in our study by impeding cell proliferation and colony formation; by facilitating apoptosis and cell cycle arrest; by decreasing cell migration and invasion; and by impairing tumor growth in vivo. Another important finding of this study is that the oncogenic effects of LINC00511 on ccRCC cells might be due to miR-625 sponging.

miRNAs exert their effects by directly interacting with the 3′-UTRs of their target mRNAs. Therefore, the mechanism by which miR-625 inhibits the progression and development of ccRCC was further illustrated in this study. First, bioinformatics prediction indicated that the 3′-UTR of *CCND1* matches the seed sequence of miR-625. Second, luciferase reporter assays demonstrated that the 3ʹ-UTR of CCND1 can be directly targeted by miR-625. Third, CCND1 was upregulated in ccRCC tissues, showing a negative correlation with miR-625 expression. Fourth, *CCND1* mRNA and protein levels were found to be reduced by miR-625 in ccRCC cells. Finally, restoration of CCND1 expression attenuated the tumor-suppressive effects of miR-625 overexpression on the malignant phenotype of ccRCC cells. These results collectively validate *CCND1* as a direct target gene of miR-625 in ccRCC.

There are several limitations in this study. First, we explored the tumor-promoting roles and the underlying molecular mechanisms of LINC00511 on A498 and 786-O cells. The functions of LINC00511 on other ccRCC cell lines need to be evaluated to bolster the roles of this lncRNA in ccRCC progression. Second, the function of LINC00511 was investigated through the downregulation of LINC00511 in a loss-of-function model. Gain-of-function studies via overexpression of LINC00511 in ccRCC cells are needed to verify our findings. We will resolve the two limitations in our subsequent experiments.

In conclusion, this study indicates that LINC00511 is upregulated in ccRCC. This upregulation closely correlates with adverse clinical characteristics and shorter overall survival. LINC00511 knockdown inhibited the malignancy of ccRCC in vitro and in vivo, partly by decreasing the competitive sponging of miR-625, which decreased CCND1 expression.

## METHODS

### Clinical tissues collection

A total of 49 pairs of ccRCC samples and matched adjacent normal renal tissue samples were collected from the patients who underwent nephrectomy at The Second Xiangya Hospital, Central South University. None of the patients had received chemotherapy or radiotherapy before surgical resection. The collected tissues were quickly frozen in liquid nitrogen and then stored at −80 °C. The study protocol was approved by the Research Ethics Committee of The Second Xiangya Hospital, Central South University. In addition, written informed consent was provided by all the participants.

### Cell culture

A normal human renal cell line (HK-2) and four ccRCC cell lines (A498, 786-O, ACHN, and Caki-2) were ordered from the American Type Culture Collection (Manassas, VA, USA). HK-2 cells were maintained in keratinocyte-SFM (Invitrogen, Carlsbad, CA, USA) containing bovine pituitary extract and human recombinant epidermal growth factor (all from Gibco, Grand Island, NY, USA). The four ccRCC cell lines were grown in Dulbecco’s modified Eagle’s medium (DMEM) with 10% (v/v) of heat-inactivated fetal bovine serum (FBS), 100 U/mL penicillin, and 100 μg/mL streptomycin (all from Gibco, Grand Island, NY, USA). All exponentially growing cells were maintained at 37 °C in a humidified incubator supplied with 5% CO_2_.

### Oligonucleotides, plasmids, and cell transfection

Small interfering RNA (siRNA) targeting LINC00511 (si-LINC00511) and negative control (NC) siRNA (si-NC) were chemically synthesized by Guangzhou Ribobio Co., Ltd. (Guangzhou, China). The miR-625 mimics, NC miRNA mimics (miR-NC), miR-625 inhibitor, and NC inhibitor were purchased from Shanghai GenePharma Co., Ltd. (Shanghai, China). To restore CCND1 expression, the full-length sequence of CCND1 without the 3′-UTR was constructed by Guangzhou GeneCopoeia Co., Ltd. (Guangzhou, China) and inserted into the pcDNA3.1 vector. The resulting construct was designated as pcDNA3.1-CCND1 (pc-CCND1).

Cells were seeded into 6-well plates at 40–50% confluence 1 day prior to transfection. Lipofectamine 2000 (Invitrogen; Thermo Fisher Scientific, Inc., Waltham, MA, USA) was used for cell transfection in accordance with the manufacturer’s protocol. Eight hours after the transfection, the cell culture medium was replaced with fresh DMEM containing 10% of FBS.

### RNA isolation and reverse-transcription quantitative polymerase chain reaction (RT-qPCR)

Total RNA was isolated from the tissues or cultured cells by means of the TRIzol reagent (Invitrogen; Thermo Fisher Scientific, Inc., Waltham, MA, USA). To quantify miR-625 expression, the miScript Reverse Transcription kit (Qiagen GmbH, Hilden, Germany) was applied to prepare complementary DNA (cDNA) from total RNA. Next, the cDNA was amplified with the miScript SYBR Green PCR kit (Qiagen GmbH, Hilden, Germany) on a Roche Light Cycler 480 Real-Time PCR System (Roche Diagnostics, Basel, Switzerland). Relative miR-625 expression was normalized to that of small nuclear RNA U6. To quantify LINC00511 and CCND1 mRNA expression, reverse transcription was performed with the PrimeScript RT Reagent Kit (Takara Biotechnology Co., Ltd., Dalian, China). After that, quantitative PCR was conducted by means of the SYBR Premix Ex Taq™ Kit (Takara Biotechnology Co., Ltd., Dalian, China). GAPDH served as endogenous control to normalize the expression levels of LINC00511 and *CCND1* mRNA. All data were analyzed by the 2^−ΔΔCt^ method.

### Cell counting kit-8 (CCK-8) assay

The proliferative ability of ccRCC cells was determined in the CCK-8 assay. At 24 h after transfection, the cells were harvested, and a single-cell suspension was prepared and seeded in 96-well plates at a density of 3,000 cells per well. Transfected cells were then incubated at 37 °C in a humidified incubator supplied with 5% CO_2_ for 0, 24, 48, or 72 h. The CCK-8 assay was performed at every time point by adding 10 μL of the CCK-8 reagent (Dojindo Molecular Technologies, Inc., Kumamoto, Japan) into each well. After another 2 h of incubation, the optical density (OD) of each well at a wavelength of 450 nm was measured on a microplate reader (Bio-Rad Laboratories, Hercules, CA, USA).

### Colony formation assay

Transfected cells were collected at 24 h post-transfection and seeded in 6-well plates at an initial density of 1000 cells/well. The cells were grown at 37 °C in a humidified incubator supplied with 5% CO_2_ for 2 weeks. On day 15, the cells were fixed with 95% methanol and then stained with methyl violet (Beyotime Institute of Biotechnology, Inc., Shanghai, China). Finally, the colonies (>50 cells) were counted under an IX71 inverted microscope (Olympus Corporation, Tokyo, Japan).

### Cell apoptosis assay

The rate of cell apoptosis was monitored using an Annexin V–Fluorescein Isothiocyanate (FITC) Apoptosis Detection Kit (Biolegend, San Diego, CA, USA). After incubation for 48 h, transfected cells were collected and washed three times with cold phosphate-buffered saline (PBS) and resuspended in 100 μL of binding buffer. Next, 5 μL of Annexin V–FITC and 5 μL of a propidium iodide solution (that came with the kit) were added to the cells and incubated at room temperature in the dark for 15 min. The apoptosis rate was determined by flow cytometry (FACScan; BD Biosciences, Franklin Lakes, NJ, USA), and the CellQuest software version 5.1 (BD Biosciences, Franklin Lakes, NJ, USA) was employed for data analysis.

### Cell cycle assay

This assay was conducted to evaluate the cell cycle status of ccRCC cells *in vitro*. In particular, transfected cells were harvested at 48 h post-transfection using EDTA-free trypsin (Gibco, Grand Island, NY, USA), washed twice with cold PBS, and then fixed in 70% ethanol at 4 °C for 1 h. The cells were centrifuged and treated with 50 μL of RNase (100 μg/mL) at room temperature for 20 min. Next, the cells were stained for 30 min at room temperature in 25 μL of the propidium iodide solution diluted in 425 μL of cell staining buffer (both from Biolegend, San Diego, CA, USA). The stained cells were subjected to flow cytometry to determine the cell cycle status.

### Transwell migration and invasion assays

These assays were carried out to assess the migration and invasion abilities of ccRCC cells using Transwell chambers (8.0 μm pore size; BD Biosciences, Franklin Lakes, NJ, USA). Transwell chambers coated with Matrigel (BD Biosciences, Franklin Lakes, NJ, USA) were used for the invasion assay. For the migration assay, no Matrigel was used. A total of 200 μL of FBS-free DMEM containing 5 × 10^4^ transfected cells was placed into the upper compartment of the Transwell chambers, while 500 μL of DMEM supplemented with 20% of FBS was added into the lower compartments to serve as a chemoattractant. Twenty-four hours later, cells remaining on the upper side of the membranes were gently wiped out with a cotton swab. The migrated and invaded cells on the other side of the membranes were fixed with 95% ethanol and stained with 0.5% crystal violet (Beyotime Institute of Biotechnology, Inc., Shanghai, China). The number of migrated and invaded cells was counted under an inverted microscope in five randomly chosen visual fields from each chamber.

### Tumor xenograft experiment

A total of eight 4-week-old BALB/c nude mice were purchased from the Shanghai Laboratory Animal Center (Chinese Academy of Sciences, Shanghai, China), and were maintained under special pathogen-free conditions. Cells transfected with si-LINC00511 or si-NC were subcutaneously administered into BALB/c nude mice. The length and width of tumor xenografts were measured using Vernier calipers every 2 days. BALB/c nude mice were euthanized at 30 days after injection, and their tumor xenografts were excised and weighed. Tumor volumes were calculated according to the following equation: tumor volume = 1/2 × tumor length × tumor width^2^. All procedures involving xenograft experiments were approved by the Ethics Review Committee of The Second Xiangya Hospital, Central South University and were carried out in accordance with the Animal Protection Law of the People’s Republic of China-2009 for experimental animals.

### Nuclear/cytoplasmic fractionation

Cytoplasmic and nuclear fractions were extracted using the PARIS Kit (Invitrogen; Thermo Fisher Scientific, Inc.) in accordance with the manufacturer’s protocols.

### RNA immunoprecipitation (RIP) assay

The binding of miR-625 to LINC00511 was detected by means of the Magna RIP RNA-Binding Protein Immunoprecipitation Kit (Millipore Inc., Billerica, MA, USA). ccRCC cells were lysed with RIP buffer. The cell extract was incubated with magnetic beads that were conjugated with a human anti-AGO2 antibody or control IgG (Millipore Inc.). After that, the collected samples were treated with proteinase K to digest the protein, followed by isolation of total RNA for RT-qPCR analysis.

### Bioinformatics prediction

starBase 3.0 (http://starbase.sysu.edu.cn/) was employed to search for the miRNAs that may be sponged by LINC00511. The putative targets of miR-625 were predicted using starBase 3.0, TargetScan (http://www.targetscan.org/), microRNA.org (http://www.microrna.org/microrna/), and miRDB (http://mirdb.org/miRDB/index.html).

### Luciferase reporter assay

The 3′-UTR fragments of *CCND1* containing the wild-type (Wt) or mutant (Mut) miR-625–binding site were produced by Shanghai GenePharma Co., Ltd., cloned into the pmirGLO luciferase reporter gene (Promega, Madison, WI, USA), and named as CCND1-Wt and CCND1-Mut, respectively. The luciferase plasmids, including LINC00511-Wt and LINC00511-Mut, were chemically generated in the same way as described above.

Cells were seeded in 24-well plates and cotransfected with the Wt or Mut luciferase reporter plasmid in the presence of the miR-625 mimics or miR-NC, using Lipofectamine 2000 according to the manufacturer’s protocol. Transfected cells were incubated at 37°C and 5% CO_2_, and harvested at 48 h after transfection. Luciferase activity was measured via a Dual-Luciferase Reporter System (Promega, Madison, WI, USA) following the manufacturer’s instructions. Firefly luciferase activity was normalized to *Renilla* luciferase activity.

### Western blot analysis

Total protein was prepared from tissues or cells using radioimmunoprecipitation assay lysis buffer (Sigma-Aldrich, St. Louis, MO, USA) containing a protease inhibitor cocktail (Promega, Madison, WI, USA). The concentration of total protein was quantified by a bicinchoninic acid assay (Beyotime Institute of Biotechnology, Inc., Shanghai, China). Equivalent amounts of protein were separated by sodium dodecyl sulfate polyacrylamide gel electrophoresis on a 10% gel and then transferred onto polyvinylidene fluoride membranes (EMD Millipore, Billerica, MA, USA). After blockage at room temperature for 2 h with 5% skim milk, the membranes were incubated with primary antibodies against CCND1 (ab16663; 1:1000 dilution; Abcam, Cambridge, UK, USA) or GAPDH (ab128915; 1:1000 dilution; Abcam, Cambridge, UK, USA). Next, a horseradish peroxidase-conjugated secondary antibody (ab205718; 1:5000 dilution; Abcam, Cambridge, UK, USA) was added and incubated at room temperature for 2 h. Finally, the immunoreactive bands were visualized with the Enhanced Chemiluminescence Reagent (Bio-Rad Laboratories, Hercules, CA, USA). GAPDH was used for normalization.

### Statistical analysis

Data are shown as the mean ± standard error. Differences between groups were analyzed by Student’s *t* test or one-way analysis of variance, followed by the Student–Newman–Keuls multiple-comparison test. The χ^2^ test was conducted to determine the relation between LINC00511 expression and clinical characteristics of ccRCC. The logrank test was conducted to analyze the association between LINC00511 expression and overall survival among the patients with ccRCC. The association between LINC00511 and miR-625 expression levels in ccRCC tissues was evaluated by Spearman’s correlation analysis. All statistical analyses were carried out in the SPSS software version 19.0 (SPSS, Inc., Chicago, IL, USA). Data with P < 0.05 were considered statistically significant.
